# Polyamines in chemiosmosis *in vivo*: A cunning mechanism for the regulation of ATP synthesis during growth and stress

**DOI:** 10.3389/fpls.2014.00071

**Published:** 2014-02-28

**Authors:** Nikolaos E. Ioannidis, Kiriakos Kotzabasis

**Affiliations:** Department of Biology, University of CreteHeraklion, Greece

**Keywords:** ATP synthesis, proton motive force, chloroplast, photosynthesis, polyamines, putrescine, stress

## Abstract

Polyamines (PAs) are low molecular weight amines that occur in every living organism. The three main PAs (putrescine, spermidine, and spermine) are involved in several important biochemical processes covered in recent reviews. As rule of thumb, increase of the cellular titer of PAs in plants is related to cell growth and cell tolerance to abiotic and biotic stress. In the present contribution, we describe recent findings from plant bioenergetics that bring to light a previously unrecognized dynamic behavior of the PA pool. Traditionally, PAs are described by many authors as organic polycations, when in fact they are bases that can be found in a charged or uncharged form. Although uncharged forms represent less than 0.1% of the total pool, we propose that their physiological role could be crucial in chemiosmosis. This process describes the formation of a PA gradient across membranes within seconds and is difficult to be tested *in vivo* in plants due to the relatively small molecular weight of PAs and the speed of the process. We tested the hypothesis that PAs act as permeable buffers in intact leaves by using recent advances *in vivo* probing. We found that an increase of PAs increases the electric component (Δψ) and decreases the ΔpH component of the proton motive force. These findings reveal an important modulation of the energy production process and photoprotection of the chloroplast by PAs. We explain in detail the theory behind PA pumping and ion trapping in acidic compartments (such as the lumen in chloroplasts) and how this regulatory process could improve either the photochemical efficiency of the photosynthetic apparatus and increase the synthesis of ATP or fine tune antenna regulation and make the plant more tolerant to stress.

## CHEMIOSMOSIS

**FIGURE 1:**
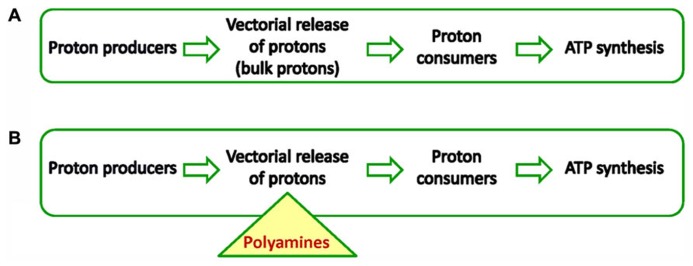
**(A)** Chemiosmosis in all cells powers ATP synthesis by forming a proton motive force. Important for the establishment of *pmf* is a membrane the so-called coupling membrane. Proton producers are usually enzymes of the respiratory chain or photosynthetic subcomplexes. Proton consumers are usually proton-driven ATPases. **(B)** New chemiosmosis. PAs buffer acidic compartment and energize the membrane that houses ATPases. The triangle shows the central point of the PAs role in chemiosmosis. In other words PAs act as intermediates receiving protons from producers and deliver them to consumers.

Organisms need ATP for many cellular processes such as translation, metabolite production, proliferation and stress response. Most ATP (95%) is produced by chemiosmosis (i.e., the movement of ions across a selectively permeable membrane, down their electrochemical gradient), therefore this synthesis is the most important process for cell physiology (Mitchell 1961, 1978). Not surprisingly, partial or full inhibition of chemiosmosis leads to disease or death in animals and plants. Hence, any factor (protein or solute) that increases or more generally speaking, modulates ATP synthesis is of exceptional biological significance. In this contribution, we will discuss the role of polyamines (PAs) in chemiosmotic ATP synthesis based on findings from plant bioenergetics. The chemiosmotic hypothesis states that ATP synthesis in respiring cells comes from the electrochemical gradient across membranes such as the inner membranes of mitochondria and chloroplasts (Kramer et al., 2004). In other words, energization of a single membrane simultaneously and continuously powers many ATP synthases. Usually in biochemistry, an enzyme converts a substrate into a product, but in chemiosmosis the situation is slightly more complex. Hence, for the purpose of this review it is important to clarify basic features of the chemiosmotic mechanism before the role of PAs is described. The chemiosmotic mechanism in plants, animals and microbes has three conserved features: (i) an electron transport chain that supports vectorial release of protons (proton producers), (ii) a coupling membrane or “energized” membrane (cristae membrane in mitochondria, thylakoid membrane in chloroplasts, and plasma membrane in bacteria), (iii) transmembrane proton motive ATPases that are vectorially embedded in the membrane (proton consumers). The following scheme (**Figure 1A**) illustrates the sequence of events in classical chemiosmosis.

A chemiosmotic unit (a membrane that houses many proton producers and many ATPases) functions as a battery and as long as it is charged phosphorylates ADP. This battery can be seen as a huge enzymatic complex that uses an electrochemical gradient also called proton motive force (*pmf*) to produce ATP. *Pmf* is a combination of two gradients across the membrane: a concentration proton gradient (ΔpH) and an electrical gradient (Δψ). In simpler terms, electron carriers and related enzymes in the membrane produce protons that are released on one side of the membrane and decrease the pH of this compartment (e.g., lumen of thylakoids). Consequently, protons will diffuse from an area of high proton concentration (lumen) to an area of lower proton concentration (stroma). The main efflux path for protons is the ATPase, which in turn uses protons’ free energy to phosphorylate ADP. Important factors for the amplitude of *pmf* are the proton release rate, the conductivity of the ATPase to protons and the ionic strength. In plants, *pmf* is established both in mitochondria and chloroplasts. Next, we will describe why *pmf *in chloroplasts has a more complex and important role than in mitochondria. Noteworthily, in plant science data derive both from *in vitro* and *in vivo* measurements. In other disciplines, most data in particular for ATPases come from *in vitro* experiments. Thus chemiosmosis *in vivo* is better understood and described in plants.

## *Pmf* IN PLANT CHLOROPLASTS

Proton motive force in chloroplasts produces energy and regulates photoprotection. Thus light-driven transthylakoid *pmf* plays several essential roles in plant physiology (Kramer et al., 2004). More particularly both the ΔpH and Δψ components of *pmf* contribute to ATP synthesis at the CF_O_–CF_1_ ATP synthase, in a thermodynamically equivalent fashion (Kramer et al., 2003). In addition, the ΔpH component of *pmf* is a key signal for initiating photoprotection. This photoprotection mechanism the so-called energization quenching (qE), is a process that harmlessly dissipates the excess absorbed light energy as heat (Li et al., 2000; Pascal et al., 2005; Ruban et al., 2007). Acidification of the lumen also controls photosynthetic electron transfer by slowing the rate of plastoquinol oxidation at the cytochrome b_6_f complex (Hope, 2000; Takizawa et al., 2007), preventing the accumulation of highly reducing species within photosystem I (Kramer and Evans, 2011).

Parsing of the thylakoid *pmf *into ΔpH and Δψ components has been observed in thylakoids (Cruz et al., 2001) and in intact leaves (Avenson et al., 2004) and was proposed to constitute an important fine-tuning mechanism for photosynthesis (Avenson et al., 2005). “Under optimal conditions, when down-regulation is not needed, a large fraction of *pmf* can be stored as Δψ, leading to moderate lumen pH and low qE, even at high *pmf* (and thus high rates of ATP synthesis). In contrast, under environmental stresses–e.g., high light, low CO_2_/O_2_, when photoprotection is advantageous–*pmf* can be predominantly stored as ΔpH, maximizing lumen acidification for a given *pmf*” (Ioannidis et al., 2012).

The mechanism by which thylakoid *pmf *is partitioned *in vivo* into Δψ and ΔpH remained until recently unclear, but *in vitro* experiments and modeling have established that at least three factors are critical (Cruz et al., 2001; Avenson et al., 2005): (i) the capacitance of the thylakoid membrane, (ii) the ionic composition of the stroma and lumen, and (iii) the proton-buffering capacity of the lumen.

We proposed that Δψ/ΔpH control involves biological weak bases, such as PAs, which occur normally in chloroplasts and can act as “permeant buffers,” specifically dissipating the ΔpH component and thus favoring Δψ (Ioannidis et al., 2012). Because the titer of these weak bases can be regulated by the organism (by synthesis, degradation, transport, conjugation, and covalent binding to proteins), this can constitute a key-mechanism for the adjustment of the ΔpH/Δψ ratio in the short (seconds) and long term (hours to days) conditions. In the following section, we explain the role of PAs in the chemiosmotic scheme. The classical scheme is expanded in order to accommodate the mode of action of PAs (**Figure 1B**).

According to Williams (1978), Mitchell also tried to incorporate data from amines (Ort et al., 1976) under similar experimental conditions to ours (Ioannidis et al., 2006) and broke the rules of chemiosmosis, expanding his theory. By that time it was not clear that amines could be used by nature in chemiosmosis, and were used as a tool to study phosphorylation. Even 10 years later Slater reviewed the numerous studies on the nature of the intermediate between the redox reaction and ATP synthesis concluding that the matter was still open (Slater, 1987). In light of recent data, biogenic amines (i.e., PAs) seem to play the role of an intermediate *in vivo,* this matter is currently being better understood.

We expand on chemiosmosis once again by introducing natural amines that their existence in thylakoids is well established and their molecular role is getting better understood. Moreover the intermediate is not obligatory for ATP synthesis as one may assume. Thus, ATP synthesis can occur *in vitro* without PAs. However, the intermediate (i.e., PAs) increases the efficiency of ATP synthesis and allows regulation (Ioannidis et al., 2006, 2012).

## THE ROLE OF POLYAMINES IN CHEMIOSMOSIS

In the past, PAs were seen by researchers as cations. This is correct to some extent, but underestimates the importance of their free forms (uncharged bases). Can free forms that are less than 0.1% of the total PA pool play a significant role in cell physiology? A widely known but rather overlooked chemical property of the PA pool is the dynamic equilibrium between the neutral base and its protonated forms. This simple property greatly increases the complexity of the PA mode of action because of ion trapping phenomena that appear when (i) a membrane barrier (for instance the thylakoid membrane) is present and (ii) a basic compartment become more acidic (acid jump) or more generally speaking a ΔpH difference is established across a membrane. PA trapping will be explained in detail below.

**FIGURE 2:**
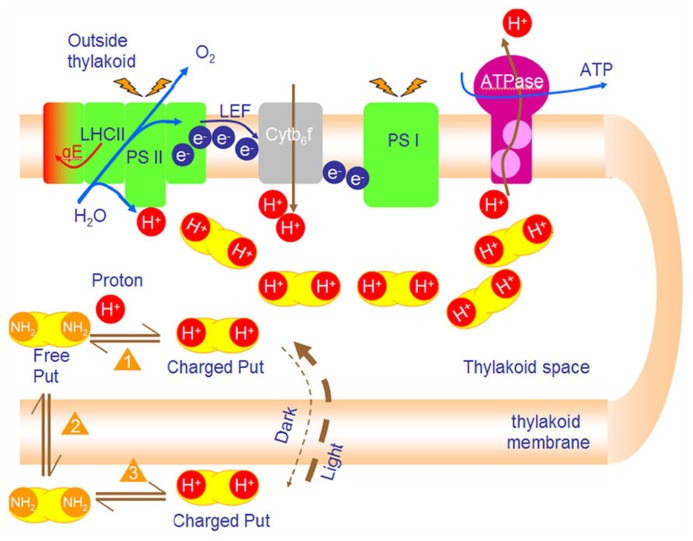
**PAs accumulate in the lumen and buffer the lumen pH during photophosphorylation**. Photosynthetic reactions produce protons that are vectorially released in the lumen. Lumen acidification shifts equilibrium 1 to the right (production of charged Put in lumen). Depletion of free putrescine urges new free Put to diffuse from stroma into lumen (Le Chatelier principle equilibrium 2). Finally, free Put in stroma is replaced by charged Put in stroma which is ionized (equilibrium 3). The net result of this process (i.e., new poise of the 3 equilibria) is ion trapping. That is the accumulation of Put in lumen up to 100 times. The final concentration of Put depends mainly on ΔpH and counterion concentration (such as Cl^-^).

A first attempt to differentiate between the effects that are due to the cationic character of PAs and those that are due to the chemical equilibrium of the free and charged forms was done in isolated thylakoids (Ioannidis et al., 2006). Cationic (coulombic) effects can induce *in vitro* stacking of thylakoids, increase photochemical efficiency of PSII and increase LEF similarly to divalent inorganic cations. Moreover, PAs can stimulate ATP synthesis in isolated thylakoids from 15 to 70% (Ioannidis and Kotzabasis, 2007). Spermine (Spm) marginally stimulated photophosphorylation (~30%) at a very low concentration (~100 μM), whereas putrescine (Put) greatly stimulated phosphorylation (~70%) at about 1000 μM (Ioannidis et al., 2006). These effects are due to the buffering role of PAs and therefore cannot be mimicked by inorganic cations. In addition, recently we verified that PAs participate in the modulation of *pmf* in thylakoids *in vivo *(Ioannidis et al., 2012). Stimulation of ATP synthesis by low molecular weight amines like imidazole, methylamine, and ammonia was formerly reported and occurs through ion trapping (Giersch and Meyer, 1984; Pick and Weiss, 1988). The term “ion trapping” is used to describe the build-up of a higher concentration of a chemical across a cell membrane due to the pK value of the chemical and difference of pH across the membrane. This results in the accumulation of basic chemicals (such as amines) in acidic compartments (such as the thylakoid lumen). In theory for a ΔpH = 2, amines inside the acidic vesicle will be 100 times more than the amines outside. PAs have relatively high pK (>7.5) and are ideal molecules for trapping phenomena in basic transitions (e.g., from pH 7 down to pH 5). The ion trapping mechanism was formerly theoretically described and experimentally demonstrated in thylakoid membranes for Put (Guarino and Cohen, 1979a, b). When chloroplasts are incubated in darkness with Put, the diamine is expected to be equally distributed at both sides of the thylakoid membrane (C_i__n_ = C_o__ut_) (Schuldiner et al., 1972). The amine in each compartment (in lumen or in stroma) is in a complex dynamic equilibrium that is demonstrated below (see equilibria 1, 2, and 3). One should pay attention to the second type of equilibrium between the permeant uncharged amine in the lumen and that in the stroma (**Figure 2**). Upon illumination protons are released in thylakoid lumen and shift the equilibrium No1 to the right. In the lumen charged amines are produced with a parallel depletion of uncharged forms (Ioannidis et al., 2006). This disequilibrium forces a rapid influx of uncharged diamines from the stroma via equilibrium No2 (let us for a moment assume that the membrane is impermeable to charged Put).

The ΔpH value defines the extent of trapping and the internal concentration of Put (C_i__n_) increases so much that the ratio C_i__n_/C_o__ut_ can be increased 500- to 3000-fold (Guarino and Cohen, 1979a, b). This gradient of Put buffers protons in the lumen, but interestingly, it does not change the total cationic charge in the lumen more than it is already increased due to proton release (Ioannidis et al., 2006). Finally, steady state ATP synthesis is stimulated. A possible cause is that the rate of proton transfer along a net of hydrogen bonds can be faster than the rate of proton transfer in water at pH 7 (Williams, 1978). Furthermore, buffering of the lumen pH by PAs keeps conditions at a more moderate pH and inhibits overacidification which in turn would hinder electron transport and photophosphorylation (Kramer et al., 2004).

**FIGURE 3:**
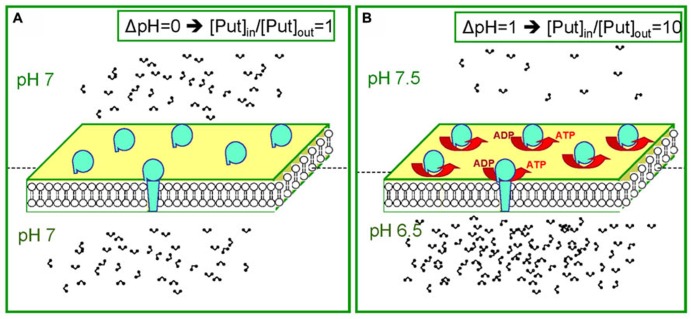
** The dual behavior of the Put pool during dark and light**. **(A)** Equal distribution of protons leads to equal distribution of PAs (for example during the dark). **(B)** Establishment of a ΔpH between the two compartments leads to unequal distribution of PAs. Note in b that for ΔpH = 1 ten times more Put will accumulate in the acidic compartment.

The assumption that thylakoids are impermeable to charged Put is an oversimplification. It is known that Cl^-^ channels of the thylakoid membrane open at 30 mV (Schoenknecht et al., 1988) and the influx of Cl^-^ (counter-ions) is expected to neutralize amine molecules and allow its efflux in the stroma (Sigalat et al., 1988). The voltage dependency of those channels may act as a safety valve sensor that hinders Put overaccumulation in the lumen and simultaneously is able to fine tune membrane potential in values sufficient for ATP synthesis (Ioannidis et al., 2006). To summarize, the data (Ioannidis et al., 2006, 2012) reveal an unknown dynamic behavior of the Put pool (dual role). We suggest that in the dark, the lumen has a relatively low concentration of Put, and in the light the lumen has a higher concentration of Put (**Figure 3**).

## POLYAMINE IMPLICATIONS IN STRESS PHYSIOLOGY WITH RESPECT TO CHEMIOSMOSIS

During the last years, plant research has focused on the role of PAs in the defense of plants against a series of environmental stress conditions (Galston, 2001); such as temperature (Hummel et al., 2004; Sfakianaki et al., 2006), salinity (Maiale et al., 2004; Demetriou et al., 2007), enhanced atmospheric ozone (Navakoudis et al., 2003) or UV-B radiation (Lütz et al., 2005; Sfichi et al., 2008). At present it is well established that many types of abiotic and biotic stress lead to an increase in the PA titer of plants and particularly of leaves. In plants, salt and osmotic stress were some of the first examples of the great increase of the PA titer (Richards and Coleman, 1952; Flores and Galston, 1984). Many reviews cover the topic (Alcázar et al., 2010a,b; Marco et al., 2011). In addition, a promising field of research is H_2_O_2_ production during stress via PA oxidases (for recent advances see Moschou and Roubelakis-Angelakis, 2011 and Moschou et al., 2008). However, the role of PAs during stress is not well understood.

New chemiosmosis may help to elucidate the role of PAs during stress. Below we consider only two cases (salt and osmotic stress) but the concept could be adopted with some modifications in other stress cases as well. “For example in *Arabidopsis* grown under high salt stress, photosynthesis would likely need to operate under conditions where the ionic strength inside the plastid is high. In this case, *pmf* storage would be heavily biased toward ΔpH formation (Robinson et al., 1983; Sacksteder and Kramer, 2000; Cruz et al., 2001). Consequently, energy dissipation would be more easily and strongly induced at low and moderate light intensities, severely limiting the productivity and growth of the plant, even if water and CO_2_ were not limiting factors. Thus, the accumulation of Put observed in plants grown under high salt stress (Alcázar et al., 2006; Bagni et al., 2006) and particularly in *Arabidopsis *through *adc2* induction (Urano et al., 2004) could serve to increase buffering solutes, rebalancing *pmf *toward Δψ and optimizing the regulation of energy transduction. In line with this view, blocking this up-regulation of Put during salt stress, e.g., in the *adc-2-1* mutant of *Arabidopsis*, leads to increased sensitivity to salt stress, which is restored upon addition of Put (Kasinathan and Wingler, 2004; Urano et al., 2004), whereas over-expressing *adc* increases tolerance to drought (Capell et al., 2004)” (Ioannidis et al., 2012). Similarly, under osmotic stress the plant faces a decrease in relative water content. This decrease in water content is also evident in chloroplasts. It is well established that under conditions of water stress arginine decarboxylase (ADC) which is located in thylakoid membranes of chloroplasts (Borrell et al., 1995) is significantly upregulated, i.e., 2–60-fold increase (Flores and Galston, 1982). For recent works of ADC up-regulation upon stress see Alcázar et al. (2010a,b). This increase of Put titer is part of the protective response of the plant to osmotic stress. Artificial increase of Put titer in leaf disk 1 h before the stress significantly protects the photosynthetic apparatus (Kotakis et al., 2014). In all cases data from leaf discs should be examined with caution. In addition, the role of other organelles such as the vacuole that contain most of the water reserves in the plant cell could be important. Hence, the titer of PAs in each compartment of the cell (e.g., chloroplasts, mitochondria, vacuole, nucleus) should be estimated both under physiological conditions and under stress. In this capacity, new protocols and methods should be used solving problems that derive from the properties of PAs (i.e., high pKs and rapid penetration of membrane barriers). PAs will accumulate *in vivo* in every cellular compartment/organelle that is more acidic than the surrounding microenviroment (the driving force is their high pK as explained before in the ion trapping) and will be depleted rapidly (within seconds) upon grinding of the tissue.

## POLYAMINE IMPLICATIONS IN GROWTH

**FIGURE 4:**
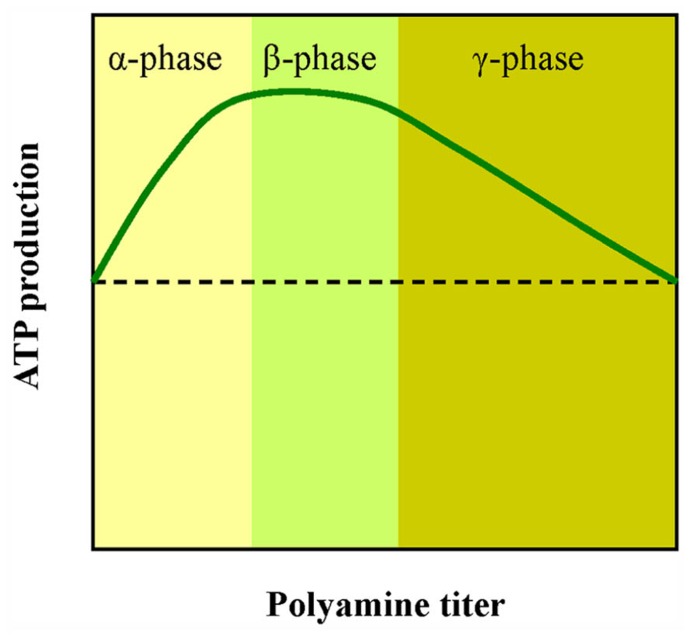
**A simplified scheme showing the effect of PAs on ATP production by thylakoids**. Low doses of PAs stimulate photophosphorylation (α phase). Higher doses lead to reduction of ATP synthesis (γ phase). The x axis is qualitative because in all three PAs (Put, Spd, and Spm) the peak value corresponds to different concentration although the shape of the curve is similar.

PAs are described in early papers as growth factors however, it is not clear why the increase of PAs stimulates growth and cell division, while inhibition of PA synthesis hinders division and growth. We suggest that these processes require a lot of ATP and the cell must boost the chemiosmotic machinery. Small changes of available Put result in different energy output under the same light energy input (phases α and **γ** in **Figure 4**). The normal Put concentration inside cells is in the fractional millimolar range, so we suggest that a small increase or decrease of available Put can fine tune ATP production. *In vivo*, lower Put content characterizes the aged cells (Paschalidis and Roubelakis-Angelakis, 2005). “This is nicely correlated with the low metabolic rates that are common during senescence. Optimal ATP rates are about 1 mM Put that might be near the value of endogenous Put of actively growing cells (β phase). In the γ phase the rates of ATP synthesis gradually decline. This curve might explain why the same amount of exogenously supplied Put can give opposite effects” (Ioannidis et al., 2006). This is a puzzling phenomenon reported often by PA researchers.

## CONSERVED FEATURES OF THE NEW CHEMIOSMOSIS AND FUTURE PERSPECTIVES

New (“polyaminylated”) chemiosmosis as presented and explained in this contribution has at least four conserved features: (i) a coupling membrane, (ii) a proton-driven ATPase, (iii) a ΔpH, and iv) a pool of free PAs (e.g., Put). These four features are parts of the *pmf* machinery in microbes, animals, and plants. Chloroplast bioenergetics was the first field to investigate this concept and we urge colleagues from other fields to check whether such phenomena occur in their systems. Former studies in mitochondria have shown that PAs can play a stimulatory role (Toninello et al., 1984; Schuber, 1989). From a biochemical point of view there is no reason for inhibition of ion trapping phenomena in mitochondria. Chloroplast bioenergetics due to recent advances can test *in vivo* such hypotheses. If PAs act similarly in other non-photosynthetic systems as permeable buffers in chemiosmotic units then many up-to-date enigmatic processes may be explained. For example the molecular role of PAs in cancer cells is not well understood although their implication in the emergence of tumors is well documented (Jänne et al., 1978). Recently, the importance of mitochondrial Δψ/pmf in cancer emergence and cancer cures was discovered (Dromparis and Michelakis, 2013).We suggest that a scientific question worth testing is whether Δψ in human cancer cells is regulated by PAs. If PAs modulate Δψ in thylakoids and in cancer cell mitochondria in a similar manner (i.e., through *pmf* modulation) then a longstanding question may finally be answered.

## Conflict of Interest Statement

The authors declare that the research was conducted in the absence of any commercial or financial relationships that could be construed as a potential conflict of interest. The Review Editor Kalliopi A. Roubelakis-Angelakis declares that, despite being affiliated to the same institution as the authors, the review process was handled objectively and no conflict of interest exists.
